# Supplementary material for: Genome Editing in Zebrafish by ScCas9 Recognizing NNG PAM
Source: Cells. 2021 Aug 16;10(8):2099. doi: 10.3390/cells10082099 (PMC8392876; doi:10.3390/cells10082099)
Supplement: Supplementary file 1 [file cells-10-02099-s001.zip › cells-1320100-supplementary.pdf]

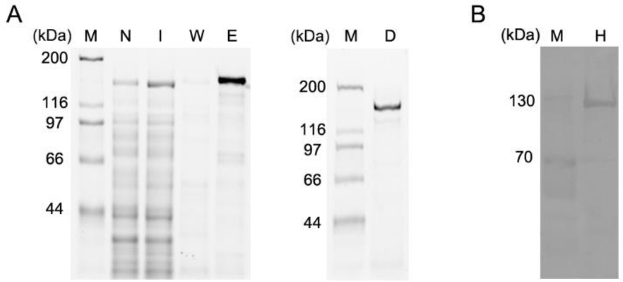

Figure. S1 Expression and purification of ScCas9 protein. **A,** The SDS-PAGE result of ScCas9 protein during enrichment and purification. M, proterin maker; N, before IPTG induction; I, after IPTG induction; W, washed fraction; E, eluted proterin; D, after dialysis in SEC buffer. **B**, The western-blot result of histone linked ScCas9. H, anti-histone antibody.

A

tyr-TAG PAM

Mutation in 6 out of 23 sequences

AGTGGACGATCGAGAGCGATGGCCTTTAGTGTTT	WT
AGTGGACGATCGAGAGCGAT - - - CTTTAGTGTTT	4/23
AGTGGACGATCGAGAGCGATGGCCGTTAATTAGTGTTT	2/23

B

tyr-CAG PAM

Mutation in 3 out of 24 sequences

CCTTTAGTGTTTTACAACCAAACCTGCCAGTG	WT
CCTTTAGTGTTTTACAACCAAACCT - - - AGTG	2/24
CCTTTAGTGTTTTACAACCAAACCTGACCAGTG	1/24

C

rpl17-NAG PAM

Mutation in 18 out of 24 sequences

ACACCCGTGAAACCGCTCAGGCCATCAAGGCATG	WT
ACACCCGTGAAACCGCTCAGGCCA - - -AGGCATG	6/24
ACACCCGTGAAACCGCTCAGGCTTTCACATCAAGGCATG	3/24
ACACCCGTGAAACCGCTCAGGAACGACAAGGCATG	2/24
- -CCATCAAGGCATG	2/24
ACACCCGTGAAACCGC - - - - - - - - - - - AAGGCATG	2/24
ACACCCGTGAAACCGCTC- GGGCATGCACAGGCATG	3/20

D

rpl9-NAG PAM

Mutation in 23 out of 24 sequences

AATGAAGACCATTCTCAGTAACCAGACAGTGGAC	WT
AATGAAGACCATTCTCAGTAACCAGTGGACAGTGGAC	5/24
AATGAAGACCATTCTCAG- - - - - - - ACAGTGGAC	3/24
AATGAAGACCATTCTCAGTAACCATCCCTGACAGTGGAC	2/24
AATGAAGACCATTCTCAGTAA - - - - - - - - - GACAGTGGAC	2/24
AATGAAGACCATTCTCAGTAACAGTGGACAGTGGAC	3/24
AATGAAGACCATTCTCAGTAAC - - - - - - - - - - - T-GAC	2/24
AATGAAGACCATTCTCAGTAAC - - - - CAGTGGAC	5/24
AATGAAGACCATTCTCAGTAAC -AGACAGTGGAC	1/24

E

rpl31-NAG PAM

Mutation in 15 out of 20 sequences

GAAGAAGGGCCGCTCGGCCATCAATGAAGTGGTG	WT
GAAGAAGGGCCGCTCGGCCATCAATGGTTTTGGAG	2/20
GAAGAAGGGCCGCTCGGGAAGGGCCGCTCGTGGTG	3/20
GAAGAAGGGCCGCTCGGCCA -C-ATGAAGTGGTG	4/20
GAAGAAGGGCCGCTCGGCCATCAGCCATGAAGTGGTG	1/20
GAAGAAGGGCCGCTCGGCCATTCTTGAAGTGGTG	2/20
GAAGAAGGGCCG - - - - - - - - - - - - - - -TGAAGTGGTG	3/20

F

ddx21-NAG PAM

Mutation in 10 out of 21 sequences

CTCCTCAACATCCAGCATGCCGTCAAAGAGTC	WT
CTCCTCAACATCCAGCATGCCGTCTCCCAAAGAGTC	2/21
CTCCTCAACATCCAGCATA - - G - - AAAGAGTC	3/21
CTCCTCAACATCCAGCATGCCGTC - - - - - - - TC	3/21
CTCCTCAACATCCAGCATGCCGTCCAGCATGCGAGTC	2/21

G

rps16-NAG PAM

Mutation in 1 out of 24 sequences

GATCAAGGATATCTTAATTCAGTACGACAGGA	WT
GATCAAGGATATCTTAATTCAGT - - - - CAGGA	1/24

H

rpl17-NTG PAM

Mutation in 11 out of 22 sequences

TCCGCAAGGCTAACAAGTACCTGAAGGATGTG	WT
TCCGCAAGGCTAAC - - - - - - - - - - AAGGATGTG	3/22
TCCGCAAGG - ATGTG	2/22
TCCGCAAGGCTAACAAGTACC - - - - - - - - - - - - -	3/22
- GGATGTG	3/22

I

rpl9-NCG PAM

Mutation in 3 out of 21 sequences

CGGTGTCCCTCAAGGGCCGCACAGTTACCGTGAAG	WT
CGGTGTC - - - - AAGGTGTTACGGAGTTACCGTGAAG	3/21

J

rpl31-NCG PAM

Mutation in 16 out of 21 sequences

TCCTTTGGCCCACACGGCCTTGTTGAGGCGTG	WT
TCCTTTGGCCCACACGGCCTTGT - - - - - - - - - G	3/21
TCCTTTGGCCCACACGGCCTTGTTTGAGGCGTG	6/21
TCCTTTGGCCCACACGGCCTTGTGTATCGAGGCGTG	3/21
TCCTTTGGCCCACACGGC - - - - - - - - - - - - - GTG	2/21
TCCTTTGGCCCACACGGCCTTGT - - - - - - - - - -	2/21

K

rps16-NCG PAM

Mutation in 7 out of 24 sequences

AGCTCGTGCTCGCTACCAGAAATCCTACCGTT	WT
AGCTCGTGCTCGCTACC------------------------GTT	3/24
AGCTCGTGCTCGCTACCAAAATCCTACCGTT	2/24
AGCTCGTGCTCGCTACCACCGTTAAAATCTCTACCACCGTT	2/24

Figure S2. Sangger sequencing result of indels induced by ScCas9 sgRNPs related to Figure 2C. **A-G**, Sequencing result of target sites with NAG PAM. **H**, Sequencing result of target sites with NTG PAM. **I-K**, Sequencing result of target sites with NCG PAM. Target site (green); PAM (red); insertion (dot) and deletion (blue).

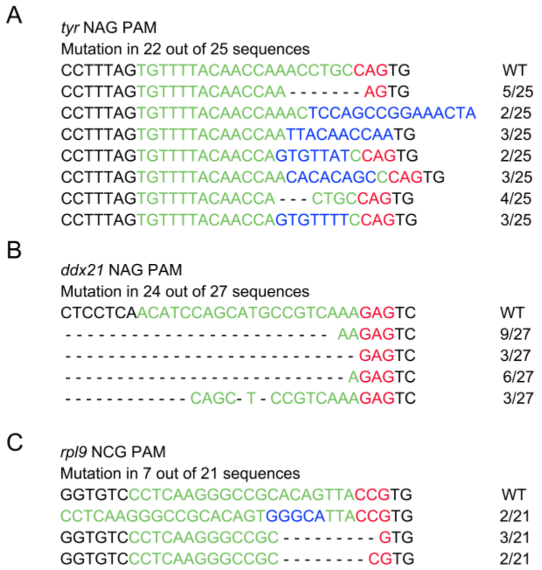

Figure S3. Sanger sequencing result of target sites induced by ScCas9 mdgRNPs related to figure 3B. **A**, Sequencing result of *tyr*-CAG site. **B**, Sequencing result of *ddx21*-NAG site. **C**, Sequencing result of *rpl9*-NCG site. Target site (green); PAM (red); insertion (dot) and deletion (blue).

Tabel S1. Nucleotide sequences of ScCas9.

1. NLS-ScCas9-NLS in pCS2+

SP6 promoter NLS SV40 terminator *Not* I M13 reverse

Star condon Stop condon

ATTTAGGTGACACTATAGAATACAAGCTACTTGTTCTTTTTGCAGGATCCATGGCCAAACGGACAGCCGACGGAAGCGAGTTCGAGTCACCAAAGAA
GAAGCGGAAAGTCGAGAAGAAATACTCCATCGGACTGGACATCGGCACCAACAGCGTGGGATGGGCCGTGATCACTGACGATTACAAAGTGCCAA
GCAAGAAATTTAAGGTGCTGGGCAACACAAACAGAAAGTCTATCAAGAAAAACCTGATGGGAGCCCTGCTGTTCGATTCCGGCGAGACCGCAGAGG
CTACTAGACTGAAGAGAACTGCAAGAAGAAGATACACAAGAAGAAAGAACAGAATCAGATACCTGCAGGAGATCTTCGCAAACGAGATGGCTAAA
CTGGACGATAGCTTCTTTCAGAGACTGGAGGAGTCTTTCCTGGTGGAGGAGGACAAGAAAAACGAGAGACACCCCATCTTTGGAAACCTGGCCGAT
GAGGTGGCATACCACAGAAACTACCCAACTATCTACCACCTGAGAAAGAAACTGGCTGATTCTCCCGAGAAGGCCGACCTGAGACTGATCTACCTGG
CCCTGGCACACATCATCAAATTCAGAGGACACTTTCTGATCGAGGGCAAGCTGAACGCAGAGAACAGCGACGTGGCTAAACTGTTCTACCAGCTGAT
CCAGACATACAACCAGCTGTTTGAGGAGTCTCCTCTGGATGAGATCGAGGTGGACGCCAAGGGAATCCTGTCCGCAAGACTGTCCAAGAGCAAAAG
ACTGGAGAAACTGATCGCAGTGTTCCCCAACGAGAAGAAAAACGGACTGTTTGGCAACATCATCGCTCTGGCCCTGGGCCTGACTCCTAACTTCAAG
AGCAACTTTGACCTGACAGAGGATGCTAAGCTGCAGCTGTCTAAAGATACTTACGACGATGACCTGGACGAGCTGCTGGGACAGATCGGCGACCAGT
ACGCCGATCTGTTCTCCGCTGCCAAAAACCTGAGCGATGCAATCCTGCTGTCTGACATCCTGAGATCTAACTCCGAGGTGACAAAAGCTCCACTGAG
CGCCTCTATGGTGAAGAGATACGATGAGCACCACCAGGACCTGGCTCTGCTGAAGACCCTTGTGAGACAGCAGTTCCCCGAGAAATACGCTGAGATC
TTTAAGGATGACACTAAAAACGGATACGCCGGCTACGTGGGAATCGGCATCAAGCACAGAAAAAGAACAACCAAGCTGGCCACACAGGAGGAGTT
CTACAAGTTTATCAAACCCATCCTGGAGAAAATGGACGGAGCAGAGGAGCTGCTGGCTAAACTGAACAGAGATGACCTGCTGAGAAAGCAGAGAA
CCTTTGATAACGGCTCTATCCCACACCAGATCCACCTGAAGGAGCTGCACGCAATCCTGAGAAGACAGGAGGAGTTCTACCCCTTTCTGAAAGAGAA
CAGAGAGAAGATCGAGAAAATCCTGACCTTCAGAATCCCTTACTACGTGGGACCACTGGCTAGAGGCAACTCTAGATTTGCCTGGCTGACAAGAAA
GTCCGAGGAGGCTATCACCCCTTGGAACTTCGAGGAGGTGGTGGACAAAGGAGCAAGCGCTCAGTCTTTCATCGAGAGAATGACTAACTTTGATGAG
CAGCTGCCCAACAAGAAAGTGCTGCCTAAGCACAGCCTGCTGTACGAGTACTTCACCGTGTACAACGAGCTGACTAAGGTGAAATACGTGACAGAG
AGAATGAGAAAACCAGAGTTCCTGTCTGGCGAGCAGAAGAAAGCCATCGTGGACCTGCTGTTTAAGACAAACAGAAAAGTGACCGTGAAGCAGCT
GAAAGAGGATTACTTCAAGAAAATCGAGTGTTTTGACTCCGTGGAAATCATCGGAGTGGAGGATAGATTTAACGCAAGCCTGGGCACATACCACGAC
CTGCTGAAGATCATCAAGGATAAAGACTTCCTGGATAACGAGGAGAACGAGGACATCCTGGAGGATATCGTGCTGACCCTGACTCTGTTTGAGGACA

GAGAGATGATCGAGGAGAGACTGAAAACATACGCCCACCTGTTCGATGACAAGGTGATGAAACAGCTGAAGAGAAGACACTACACCGGATGGGGC
AGACTGTCCAGAAAGATGATCAACGGAATCAGAGATAAGCAGAGCGGCAAAACCATCCTGGATTTTCTGAAATCCGACGGATTCAGCAACAGAAAC
TTTATGCAGCTGATCCACGATGACTCCCTGACTTTCAAGGAGGAGATCGAGAAAGCTCAGGTGTCCGGACAGGGCGATAGCCTGCACGAGCAGATCG
CAGACCTGGCTGGAAGCCCCGCCATCAAGAAAGGCATCCTGCAGACCGTGAAGATCGTGGACGAGCTGGTGAAGGTGATGGGACACAAACCTGAG
AACATCGTGATCGAGATGGCTAGAGAGAACCAGACTACAACCAAAGGACTGCAGCAGAGCAGAGAGAGAAAGAAAAGAATCGAGGAGGGCATCA
AAGAGCTGGAGTCTCAGATCCTGAAGGAGAACCCTGTGGAGAACACACAGCTGCAGAACGAGAAGCTGTACCTGTACTACCTGCAGAACGGAAGA
GACATGTACGTGGATCAGGAGCTGGACATCAACAGACTGTCCGATTACGACGTGGATCACATCGTGCCTCAGTCCTTTATCAAAGATGACAGCATCGA
CAACAAGGTGCTGACCAGAAGCGTGGAGAACAGGGGCAAGTCTGATAACGTGCCATCCGAGGAGGTGGTGAAGAAAATGAAAAACTACTGGAGAC
AGCTGCTGAACGCAAAGCTGATCACTCAGAGAAAATTTGACAACCTGACAAAGGCTGAGAGAGGAGGCCTGTCTGAGGCCGATAAGGCAGGATTC
ATCAAAAGACAGCTGGTGGAGACAAGACAGATCACCAAGCACGTGGCCAGAATCCTGGACTCCAGAATGAACACTAAAAGAGACAAGAACGATA
AACCAATCAGAGAGGTGAAAGTGATCACACTGAAGTCTAAACTGGTGTCCGATTTCAGAAAAGACTTTCAGCTGTACAAAGTGAGAGACATCAACA
ACTACCACCACGCTCACGATGCCTACCTGAACGCCGTGGTGGGAACCGCACTGATCAAGAAATACCCCAAGCTGGAGTCTGAGTTCGTGTACGGCGA
TTACAAAGTGTACGACGTGAGAAAGATGATCGCTAAATCTGAGCAGGAGATCGGAAAGGCCACAGCAAAGAGATTCTTTTACTCCAACATCATGAAC
TTCTTTAAGACCGAGGTGAAACTGGCCAACGGCGAGATCAGAAAGAGACCCCTGATCGAGACTAACGGAGAGACAGGCGAGGTGGTGTGGAACAA
GGAGAAAGACTTTGCAACCGTGAGAAAGGTGCTGGCTATGCCTCAGGTGAACATCGTGAAGAAAACCGAGGTGCAGACTGGAGGCTTCTCCAAAG
AGAGCATCCTGTCTAAGAGAGAGAGCGCCAAACTGATCCCTAGAAAGAAAGGATGGGATACCAGAAAGTACGGAGGCTTTGGCTCTCCAACTGTGG
CCTACTCCATCCTGGTGGTGGCAAAAGTGGAGAAGGGAAAAGCTAAGAAACTGAAGAGCGTGAAAGTGCTGGTGGGAATCACTATCATGGAGAAG
GGCTCTTACGAGAAAGACCCTATCGGATTCCTGGAGGCTAAGGGCTACAAAGATATCAAGAAAGAGCTGATCTTCAAGCTGCCAAAATACTCCCTGT
TTGAGCTGGAGAACGGAAGAAGAAGAATGCTGGCTAGCGCCACTGAGCTGCAGAAAGCAAACGAGCTGGTGCTGCCACAGCACCTTGTGAGACTG
CTGTACTACACACACAGAACATCAGCGCTACTACAGGATCTAACAACCTGGGCTACATCGAGCAGCACAGAGAGGAGTTCAAGGAGATCTTTGAGAAA
ATCATCGACTTCAGCGAGAAGTACATCCTGAAGAACAAAGTGAACTCTAACCTGAAAAGCTCTTTCGATGAGCAGTTTGCCGTGTCCGACAGCATCC
TGCTGTCTAACTCCTTCGTGAGCCTGCTGAAGTACACAAGCTTTGGAGCATCTGGAGGCTTCACCTTTCTGGACCTGGATGTGAAACAGGGCAGACTG
AGATACCAGACAGTGACCGAGGTGCTGGATGCTACCCTGATCTACCAGTCTATCACTGGACTGTACGAGACTAGAACAGATCTGTCCCAGCTGGGAG
GCGACAAACGGACAGCCGACGGAAGCGAGTTCGAGTCACCAAAGAAGAAGCGGAAAGTCTGACTCGAGCCTCTAGAACTATAGTGAGTCGTATTA
CGTAGATCCAGACATGATAAGATACATTGATGAGTTTGGACAAACCACAACTAGAATGCAGTGAAAAAAATGCTTTATTTGTGAAATTTGTGATGCTAT
TGCTTTATTTGTAACCATTATAAGCTGCAATAAACAAGTTAACAACAACAATTGCATTCATTTTATGTTTCAGGTTCAGGGGGAGGTGTGGGAGGTTTTT

TAATTCGCGGCCGCGGCGCCAATGCATTGGGCCCGGTACCCAGCTTTTGTTCCCTTTAGTGAGGGTTAATTGCGCGCTTGGCGTAATCATGGTCATAGC
TGTTTCCTG

2. NLS-ScCas9-NLS in pET-28b

T7 Promoter NLS 6X His T7 terminator Star condon Stop condon

TAATACGACTCACTATAGGGGAATTGTGAGCGGATAACAATTCCCCTCTAGAAATAATTTTGTTTAACTTTAAGAAGGAGATATACCATGGCCAAACGG
ACAGCCGACGGAAGCGAGTTCGAGTCACCAAAGAAGAAGCGGAAAGTCGAGAAGAAATACTCCATCGGACTGGACATCGGCACCAACAGCGTGG
GATGGGCCGTGATCACTGACGATTACAAAGTGCCAAGCAAGAAATTTAAGGTGCTGGGCAACACAAACAGAAAGTCTATCAAGAAAAACCTGATGG
GAGCCCTGCTGTTCGATTCCGGCGAGACCGCAGAGGCTACTAGACTGAAGAGAACTGCAAGAAGAAGATACACAAGAAGAAAGAACAGAATCAGA
TACCTGCAGGAGATCTTCGCAAACGAGATGGCTAAACTGGACGATAGCTTCTTTCAGAGACTGGAGGAGTCTTTCCTGGTGGAGGAGGACAAGAAA
AACGAGAGACACCCCATCTTTGGAAACCTGGCCGATGAGGTGGCATACCACAGAAACTACCCAACTATCTACCACCTGAGAAAGAAACTGGCTGAT
TCTCCCGAGAAGGCCGACCTGAGACTGATCTACCTGGCCCTGGCACACATCATCAAATTCAGAGGACACTTTCTGATCGAGGGCAAGCTGAACGCAG
AGAACAGCGACGTGGCTAAACTGTTCTACCAGCTGATCCAGACATACAACCAGCTGTTTGAGGAGTCTCCTCTGGATGAGATCGAGGTGGACGCCAA
GGGAATCCTGTCCGCAAGACTGTCCAAGAGCAAAAGACTGGAGAAACTGATCGCAGTGTTCCCCAACGAGAAGAAAAACGGACTGTTTGGCAACA
TCATCGCTCTGGCCCTGGGCCTGACTCCTAACTTCAAGAGCAACTTTGACCTGACAGAGGATGCTAAGCTGCAGCTGTCTAAAGATACTTACGACGAT
GACCTGGACGAGCTGCTGGGACAGATCGGCGACCAGTACGCCGATCTGTTCTCCGCTGCCAAAAACCTGAGCGATGCAATCCTGCTGTCTGACATCC
TGAGATCTAACTCCGAGGTGACAAAAGCTCCACTGAGCGCCTCTATGGTGAAGAGATACGATGAGCACCACCAGGACCTGGCTCTGCTGAAGACCCT
TGTGAGACAGCAGTTCCCCGAGAAATACGCTGAGATCTTTAAGGATGACACTAAAAACGGATACGCCGGCTACGTGGGAATCGGCATCAAGCACAG
AAAAAGAACAACCAAGCTGGCCACACAGGAGGAGTTCTACAAGTTTATCAAACCCATCCTGGAGAAAATGGACGGAGCAGAGGAGCTGCTGGCTA
AACTGAACAGAGATGACCTGCTGAGAAAGCAGAGAACCTTTGATAACGGCTCTATCCCACACCAGATCCACCTGAAGGAGCTGCACGCAATCCTGA
GAAGACAGGAGGAGTTCTACCCCTTTCTGAAAGAGAACAGAGAGAAGATCGAGAAAATCCTGACCTTCAGAATCCCTTACTACGTGGGACCACTGG
CTAGAGGCAACTCTAGATTTGCCTGGCTGACAAGAAAGTCCGAGGAGGCTATCACCCCTTGGAACTTCGAGGAGGTGGTGGACAAAGGAGCAAGCG
CTCAGTCTTTCATCGAGAGAATGACTAACTTTGATGAGCAGCTGCCCAACAAGAAAGTGCTGCCTAAGCACAGCCTGCTGTACGAGTACTTCACCGT
GTACAACGAGCTGACTAAGGTGAAATACGTGACAGAGAGAATGAGAAAACCAGAGTTCCTGTCTGGCGAGCAGAAGAAAGCCATCGTGGACCTGC
TGTTTAAGACAAACAGAAAAGTGACCGTGAAGCAGCTGAAAGAGGATTACTTCAAGAAAATCGAGTGTTTTGACTCCGTGGAAATCATCGGAGTGG
AGGATAGATTTAACGCAAGCCTGGGCACATACCACGACCTGCTGAAGATCATCAAGGATAAAGACTTCCTGGATAACGAGGAGAACGAGGACATCC

TGGAGGATATCGTGCTGACCCTGACTCTGTTTGAGGACAGAGAGATGATCGAGGAGAGACTGAAAACATACGCCCACCTGTTCGATGACAAGGTGAT
GAAACAGCTGAAGAGAAGACACTACACCGGATGGGGCAGACTGTCCAGAAAGATGATCAACGGAATCAGAGATAAGCAGAGCGGCAAAACCATC
CTGGATTTTCTGAAATCCGACGGATTCAGCAACAGAAACTTTATGCAGCTGATCCACGATGACTCCCTGACTTTCAAGGAGGAGATCGAGAAAGCTC
AGGTGTCCGGACAGGGCGATAGCCTGCACGAGCAGATCGCAGACCTGGCTGGAAGCCCCGCCATCAAGAAAGGCATCCTGCAGACCGTGAAGATC
GTGGACGAGCTGGTGAAGGTGATGGGACACAAACCTGAGAACATCGTGATCGAGATGGCTAGAGAGAACCAGACTACAACCAAAGGACTGCAGCA
GAGCAGAGAGAGAAAGAAAAGAATCGAGGAGGGCATCAAAGAGCTGGAGTCTCAGATCCTGAAGGAGAACCCTGTGGAGAACACACAGCTGCAG
AACGAGAAGCTGTACCTGTACTACCTGCAGAACGGAAGAGACATGTACGTGGATCAGGAGCTGGACATCAACAGACTGTCCGATTACGACGTGGAT
CACATCGTGCCTCAGTCCTTTATCAAAGATGACAGCATCGACAACAAGGTGCTGACCAGAAGCGTGGAGAACAGGGGCAAGTCTGATAACGTGCCA
TCCGAGGAGGTGGTGAAGAAAATGAAAAACTACTGGAGACAGCTGCTGAACGCAAAGCTGATCACTCAGAGAAAATTTGACAACCTGACAAAGGC
TGAGAGAGGAGGCCTGTCTGAGGCCGATAAGGCAGGATTCATCAAAAGACAGCTGGTGGAGACAAGACAGATCACCAAGCACGTGGCCAGAATCC
TGGACTCCAGAATGAACACTAAAAGAGACAAGAACGATAAACCAATCAGAGAGGTGAAAGTGATCACACTGAAGTCTAAACTGGTGTCCGATTTCA
GAAAAGACTTTCAGCTGTACAAAGTGAGAGACATCAACAACTACCACCACGCTCACGATGCCTACCTGAACGCCGTGGTGGGAACCGCACTGATCA
AGAAATACCCCAAGCTGGAGTCTGAGTTCGTGTACGGCGATTACAAAGTGTACGACGTGAGAAAGATGATCGCTAAATCTGAGCAGGAGATCGGAA
AGGCCACAGCAAAGAGATTCTTTTACTCCAACATCATGAACTTCTTTAAGACCGAGGTGAAACTGGCCAACGGCGAGATCAGAAAGAGACCCCTGA
TCGAGACTAACGGAGAGACAGGCGAGGTGGTGTGGAACAAGGAGAAAGACTTTGCAACCGTGAGAAAGGTGCTGGCTATGCCTCAGGTGAACATC
GTGAAGAAAACCGAGGTGCAGACTGGAGGCTTCTCCAAAGAGAGCATCCTGTCTAAGAGAGAGAGCGCCAAACTGATCCCTAGAAAGAAAGGATG
GGATACCAGAAAGTACGGAGGCTTTGGCTCTCCAACTGTGGCCTACTCCATCCTGGTGGTGGCAAAAGTGGAGAAGGGAAAAGCTAAGAAACTGAA
GAGCGTGAAAGTGCTGGTGGGAATCACTATCATGGAGAAGGGCTCTTACGAGAAAGACCCTATCGGATTCCTGGAGGCTAAGGGCTACAAAGATATC
AAGAAAGAGCTGATCTTCAAGCTGCCAAAATACTCCCTGTTTGAGCTGGAGAACGGAAGAAGAAGAATGCTGGCTAGCGCCACTGAGCTGCAGAAA
GCAAACGAGCTGGTGCTGCCACAGCACCTTGTGAGACTGCTGTACTACACACAGAACATCAGCGCTACTACAGGATCTAACAACCTGGGCTACATCG
AGCAGCACAGAGAGGAGTTCAAGGAGATCTTTGAGAAAATCATCGACTTCAGCGAGAAGTACATCCTGAAGAACAAAGTGAACTCTAACCTGAAA
AGCTCTTTCGATGAGCAGTTTGCCGTGTCCGACAGCATCCTGCTGTCTAACTCCTTCGTGAGCCTGCTGAAGTACACAAGCTTTGGAGCATCTGGAGG
CTTCACCTTTCTGGACCTGGATGTGAAACAGGGCAGACTGAGATACCAGACAGTGACCGAGGTGCTGGATGCTACCCTGATCTACCAGTCTATCACTG
GACTGTACGAGACTAGAACAGATCTGTCCCAGCTGGGAGGCGACAAACGGACAGCCGACGGAAGCGAGTTCGAGTCACCAAAGAAGAAGCGGAA
AGTCCTCGAGCACCACCACCACCACCACTGAGATCCGGCTGCTAACAAAGCCCGAAAGGAAGCTGAGTTGGCTGCTGCCACCGCTGAGCAATAACT
AGCATAACCCCTTGGGGCCTCTAAACGGGTCTTGAGGGGTTTTTTGCTGAAAGGAGGAACTATATCCG

Tabel S2. All the primers used in the study.

Gene	Sequence of the primer (5'-3')
rpl9	F: CCATTGAGGCGACTCGAGAA<br>R:TGCATTTTAACAACTCCAGCAAGA
rpl31(exon2 detect)	F: ACGTTCAGTGTGTCTGTTGT<br>R: AACATGCGGCAATCTATTCTGT
rpl31(exon3 detect)	F: TGTAAACGCAGCTCCTCTGT<br>R: CTACCGTGTGCTTTCTGCCG
rpl17	F: TTTGGTCACATGGCTGCCTAATA<br>R: CTAATGGCTTTACCACCCACAAA
ddx21	F: CCATTTGGGACGCAGCTATG<br>R: CACGTGACCACACACGTCAT
tyr	F: CGTCTCCGGTGTGTGTGAAG<br>R:CTGATGTTGGCGAACATTGGC
rps16	F: CAGCAAATTAAGAAATGTACGAGGT<br>R: TGAACGACAGTTTAGCGCTTT
ScCas9 into pCS2+	F: AGCTACTTGTTCTTTTTGCAggatccATGGCCAAACGGACAGCCGACGGAAG

	R: ACTCACTATAGTTCTAGAGGctcgagTCAGACTTTCCGCTTCTTCTTTGGTGAC
ScCas9 into pET-28b	F: TTAAGAAGGAGATATACCATGGCCAAACGGACAGCCGACGGAAGCGAGTT<br>R: GATCTCAgtggtggtggtggtggtgCTCGAGGACTTTCCGCTTCTTCTTTGGTG
ScCas9 gRNA scaffold	GTTTTAGAGCTAGGTACCTAGCAAGTTAAAATAAGGCTAGTCCGTTATCAACT<br>TGAAAAAGTGGCACCGAGTCGGTGCTTTTT
SpCas9 gRNA scaffold	GTTTTAGAGCTAGAAATAGCAAGTTAAAATAAGGCTAGTCCGTTATCAACTTG<br>AAAAAGTGGCACCGAGTCGGTGCTTTTTTT

Tabel S3. All the target sites of ScCas9.

PAM	Gene	Target sequence (N20+PAM)	Mutation frequency(Ratio, %)
NAG	*rps16*	GATATCTTAATTCAGTACGACAGGA	1/24, 4.2%
	Tyr-1	CGATCGAGAGCGATGGCCTTTAGTG	6/23, 26.1%
	Tyr-2	TGTTTTACAACCAAACCTGCCAGTG	3/24, 12.5%
	rpl17	GTGAAACCGCTCAGGCCATCAAGGG	18/24, 75%
	rpl9	GACCATTCTCAGTAACCAGACAGTG	23/24, 95.8%
	rpl31	GGGCCGCTCGGCCATCAATGAAGTG	15/20, 75%
	ddx21	ACATCCAGCATGCCGTCAAAGAGTC	10/21, 47.6%
NCG	*ddx21*	GCTCCTCAACATCCAGCATGCCGTC	0/24, 0%
	rps16	GCTCGCTACCAGAAATCCTACCGTT	7/24, 29.2%
	rpl31	ACCACTTCATTGATGGCCGAGCGGC	16/21, 76.2%
	rpl17	AAGCACCAGTGTGTTCCCTTCCGTC	0/23, 0%
	rpl9	CCTCAAGGGCCGCACAGTTACCGTG	3/21, 14.3%
NTG	*ddx21*	GCCGCTCCTCAACATCCAGCATGCC	0/23, 0%
	rpl31	GAAGGGCCGCTCGGCCATCAATGAA	0/23, 0%
	rpl17	GGCTAACAAGTACCTGAAGGATGTG	11/22, 50%
	rpl9	GACAGTGGACATCCCTGACAATGGT	0/21, 0%
	rps16	CGACAGGACCCTGCTGGTTGCTGAT	0/23, 0%
	tyr	ATCGAGAGCGATGGCCTTTAGTGTT	0/20, 0%

Tabel S4. Germline targeting efficiency and germline transmission rate of ScCas9-induced indels in zebrafish

Gene	Germline targeting efficiency	Germline transmission rate
tyr-TAG	100% (8/8)	1#, 41.7% (10/24)
rpl9-NAG	75% (6/8)	4#, 54.2% (13/24)
